# The ball and stick neuron model accounts both for microscopic and macroscopic power laws

**DOI:** 10.1186/1471-2202-12-S1-P91

**Published:** 2011-07-18

**Authors:** Klas H Pettersen, Henrik Lindén, Tom Tetzlaff, Gaute T Einevoll

**Affiliations:** 1 Department of Mathematical Sciences and Technology, Norwegian University of Life Sciences, Ås, 1432, Norway

## 

Ever since Hans Berger recorded the first human electroencephalogram (EEG) in 1924 [1] its features have been under extensive study, especially since many of them are directly related to disease and to states of consciousness. In the last decades, the underlying background spectra (the power spectral density, PSD) of the EEG have also attracted great attention. The PSD is often well fit by a 1/f^α^ power law, with α typically in the range from 1 to 2 [2,3].

Linking features seen in global recordings, such as the EEG, to features in the underlying local activity, such as single neuron activity, is still a major challenge within the field of neuroscience. Power laws are, however, recorded both at the macroscopic level, e.g., for the EEG or the MEG, and at the level of single neurons. The PSD of the sub-threshold soma potential has been shown to exhibit a 1/f^α^ power law, but with a larger power α than for the EEG. For the sub-threshold soma potential α is typically ranging from 2 to 3 [4-8]. As for the EEG, this power law seems to be very robust; it has been observed in single neuron recordings across several species and brain regions, and also in cell cultures [4-8].

Here, we present analytical solutions to the ball and stick neuron model with input currents spread homogeneously throughout the dendritic stick. Expressions for the PSD of the soma potential and the PSD of the single neuron contribution to the EEG are derived and shown to follow 1/f^α^ power laws with values of α in agreement with experiments. The scale-free background spectra of the EEG may therefore originate from stochastic processes within the neuronal membrane, rather than relying on complicated network dynamics or self-critical states.
